# Antibiotic resistance pattern of *Bacteroides fragilis* isolated from clinical and colorectal specimens

**DOI:** 10.1186/s12941-021-00435-w

**Published:** 2021-04-23

**Authors:** Seyedesomaye Jasemi, Mohammad Emaneini, Zahra Ahmadinejad, Mohammad Sadegh Fazeli, Leonardo A. Sechi, Fatemah Sadeghpour Heravi, Mohammad Mehdi Feizabadi

**Affiliations:** 1 Department of Microbiology, School of Medicine, Tehran University of Medical Sciences, Poursina Street, Engelab-e-Eslami Avenue, Tehran, Iran; 2 Department of Infectious Diseases, Imam Khomeini Hospital Complex, Tehran University of Medical Sciences, Tehran, Iran; 3 Department of Surgery, Imam Khomeini Hospital Complex, Tehran University of Medical Sciences, Tehran, Iran; 4 Department of Biomedical Sciences, University of Sassari, Sassari, Italy; 5 Surgical Infection Research Group, Faculty of Medicine and Health Sciences, Macquarie University, Sydney, Australia

**Keywords:** *Bacteroides fragilis*, Antibiotic resistance, Resistance gene, *bft* gene

## Abstract

**Background:**

*Bacteroides fragilis* is a part of the normal gastrointestinal flora, but it is also the most common anaerobic bacteria causing the infection. It is highly resistant to antibiotics and contains abundant antibiotic resistance mechanisms.

**Methods:**

The antibiotic resistance pattern of 78 isolates of *B. fragilis* (22 strains from clinical samples and 56 strains from the colorectal tissue) was investigated using agar dilution method. The gene encoding *Bacteroides fargilis* toxin *bft*, and antibiotic resistance genes were targeted by PCR assay.

**Results:**

The highest rate of resistance was observed for penicillin G (100%) followed by tetracycline (74.4%), clindamycin (41%) and cefoxitin (38.5%). Only a single isolate showed resistance to imipenem which contained *cfiA* and *IS1186* genes. All isolates were susceptible to metronidazole. Accordingly, *tetQ* (87.2%), *cepA* (73.1%) and *ermF* (64.1%) were the most abundant antibiotic-resistant genes identified in this study. MIC values for penicillin, cefoxitin and clindamycin were significantly different among isolates with the *cepA*, *cfxA* and *ermF* in compare with those lacking such genes. In addition, 22.7 and 17.8% of clinical and GIT isolates had the *bft* gene, respectively.

**Conclusions:**

The finding of this study shows that metronidazole is highly in vitro active agent against all of *B. fragilis* isolates and remain the first-line antimicrobial for empirical therapy.

## Background

*Bacteroides fragilis* is an anaerobic, Gram-negative bacteria and a part of the human gastrointestinal microbiota but can cause opportunistic infections in human. The genus *Bacteroides* accounts for about 25% of gastrointestinal tract (GIT) flora [1, 2]. Among various species of this genus and other endogenous anaerobic bacteria, *Bacteroides fragilis* (*B. fragilis*) has also been found as the most virulent and abundant opportunistic anaerobic bacterium isolated from clinical specimens [1, 3]. *B. fragilis* represents 1–2% of the normal flora of the gastrointestinal tract and, if dislocated into other anatomical sites, cause various infections such as abdominal infections, abscesses, skin and soft tissue infection, and bacteremia with a mortality rate of about 19% [1, 4]. Studies have further established that the enterotoxigenic *B. fragilis* (ETBF) strains are more pathogenic than non-toxigenic (NTBF) ones and they are associated with various diseases such as septicaemia, diarrhoea, irritable bowel syndrome (IBS), and colorectal cancer (CRC) [5, 6].

Several studies have further revealed that *B. fragilis* exhibits the highest antibiotic resistance and the most numerous antibiotic resistance mechanisms compared with other anaerobic bacteria in the GIT [7]. This not only makes it difficult to treat infections caused by *B. fragilis*, but also has the potential to act as a reservoir of antibiotic-resistant genes [8], leading to their transfer to other normal bacterial flora through integrated transposons, integrated genetic elements, as well as conjugative plasmids [9].

In this respect, different resistance patterns of this bacterium have been so far reported from different parts of the world. There have been reports of increased resistance to carbapenems and beta-lactams among *B. fragilis* isolates worldwide [6, 10–13]. Of note, the rate of resistance to metronidazole, as an effective antibiotic against anaerobic bacteria, is about 1%, but some reference laboratories have reported a resistance rate of up to 7.5% [14–16]. Moreover, the number of multidrug-resistant *B. fragilis* isolates has increased over the last decade [17–19]. Improper and excessive use of antibiotics without a doctor’s supervision are the reason for promotes bacterial resistance [20].

Despite this growing problem, few studies are reported about the rate of antibiotic resistance and the accordance of antibiotic resistance genes among *B.fragilis* strains from Iran. Therefore, in this study antibiotic resistance profiles of *B. fragilis* isolated from the GIT and clinical samples were evaluated using phenotypic and genotypic methods.

## Materials and methods

### Study population

The current cross-sectional study examined two populations, the patients, and the healthy controls. This study was approved by the Ethics Committee of National Institute for Medical Research development in Iran (NO. 971329). Informed consent was obtained from all individual participants.

The patient population included people suspected of having anaerobic infection hospitalized in different wards of Imam Khomeini Hospital of Tehran, and the healthy population included people with no history of GIT disease or antibiotic consumption in the past 3 months.

In the sampling process from the patients, 130 different clinical samples were collected from hospitalized patients in different wards of the hospital during 1 year (from August 2018 to August 2019). Sampling, culture and isolation of anaerobic bacteria were performed according to standard procedures [21].

In the sampling process from healthy individuals, 40 biopsies of the colorectal were collected by a physician during colonoscopy. To isolate *B. fragilis*, the biopsy sample was homogenized by mortar and pestle, and then 2–3 drops were inoculated on a plate containing Bacteroides Bile Esculin Agar (BBE) and Brucella Blood Agar (BBA) containing 5% sheep blood, vitamin K1 (0.5 mg/L) and hemin (5 mg/L) and cultured by isolation method. The cultivated plates were incubated for 48–72 h at 37 °C under anaerobic conditions. The black-colored colonies on the BBE medium and the ones grown on the BBA medium (5–10 colonies) were subcultured on the BBA medium. Ultimately, after confirming the phenotypic features (growth only in anaerobic conditions, Gram morphology, positivity to esculin and catalase), the isolates were preserved at − 80 °C using 5% glycerol [21, 22].

.

### **Identification of*B. fragilis***

The anaerobic bacteria were phenotypically identified based on colony morphology, Gram staining, and differential tests such as catalase, indole, bile disc, and finally Vitek 2 system (Biomerieux, France). Two polymerase chain reactions (PCR) were also performed to amplify the 16 S rDNA gene fragment; the first reaction to confirm the *B. fragilis* group and the second reaction the *B. fragilis* species [23, 24]. The 16 S rDNA gene was sequenced for *B. fragilis* strains and then submitted to the GenBank sequence database.

### **Antibiotic susceptibility of*B. fragilis*isolates**

The antibiotic susceptibility testing of *B. fragilis* isolates was performed by agar dilution method according to the Clinical and Laboratory Standards Institute (CLSI) guidelines [25]. The tested antibiotics included ampicillin/sulbactam, piperacillin/tazobactam, penicillin G, tetracycline, imipenem, meropenem, clindamycin, cefoxitin, and metronidazole. Different concentrations of the antibiotics were included in the BBA medium containing vitamin K1 (0.5 mg/l) and hemin (5 mg/l).

Moreover, 10 µl of microbial suspension with a density of 10^7^ colony-forming unit (CFU) ml^− 1^ was placed onto the plates containing antibiotics to achieve a final dilution of 10^5^ CFU per spot. Plate without the antibiotic or the bacterial suspension, was used as negative controls (NC).

The plates were also incubated for 48 h at 36 ºC under anaerobic conditions. The lowest antibiotic concentration that inhibits the appearance of bacterial growth, was determined as the MIC (Minimum inhibitor concentration). Resistance levels to different antibiotics obtained via the breakpoints recommended by the CLSI.

### Identification of resistance genes

The presence of IS1186 and *cfiA* gene (associated with resistance to carbapenems), the *cepA* and *cfxA* genes (associated with resistance to beta-lactams), the *ermF*, *ermB*, and *mefA* genes (associated with resistance to clindamycin), the *tetQ* gene (associated with resistance to tetracycline) and the *nim* gene (associated with resistance to metronidazole) were determined by PCR in *B. fragilis* isolates [26]. In order to detect the *bft* gene using PCR, parts of this gene were amplified [27].

### Statistical analysis

Data were analysed using the SPSS ver. 18.0 (SPSS Inc., Chicago, IL). The Chi-square test was performed to calculate significant differences between the presence of antibiotic resistance genes among resistant strains in comparison to non-resistant strains. Also, Mann-Whitney test was applied to examine significant differences of MIC value for each antibiotic class among isolates with resistance genes in compare with isolates lacking these genes. A *p*-value less than 0.05 was considered as statistically significant.

## Results

**Fig. 1:**
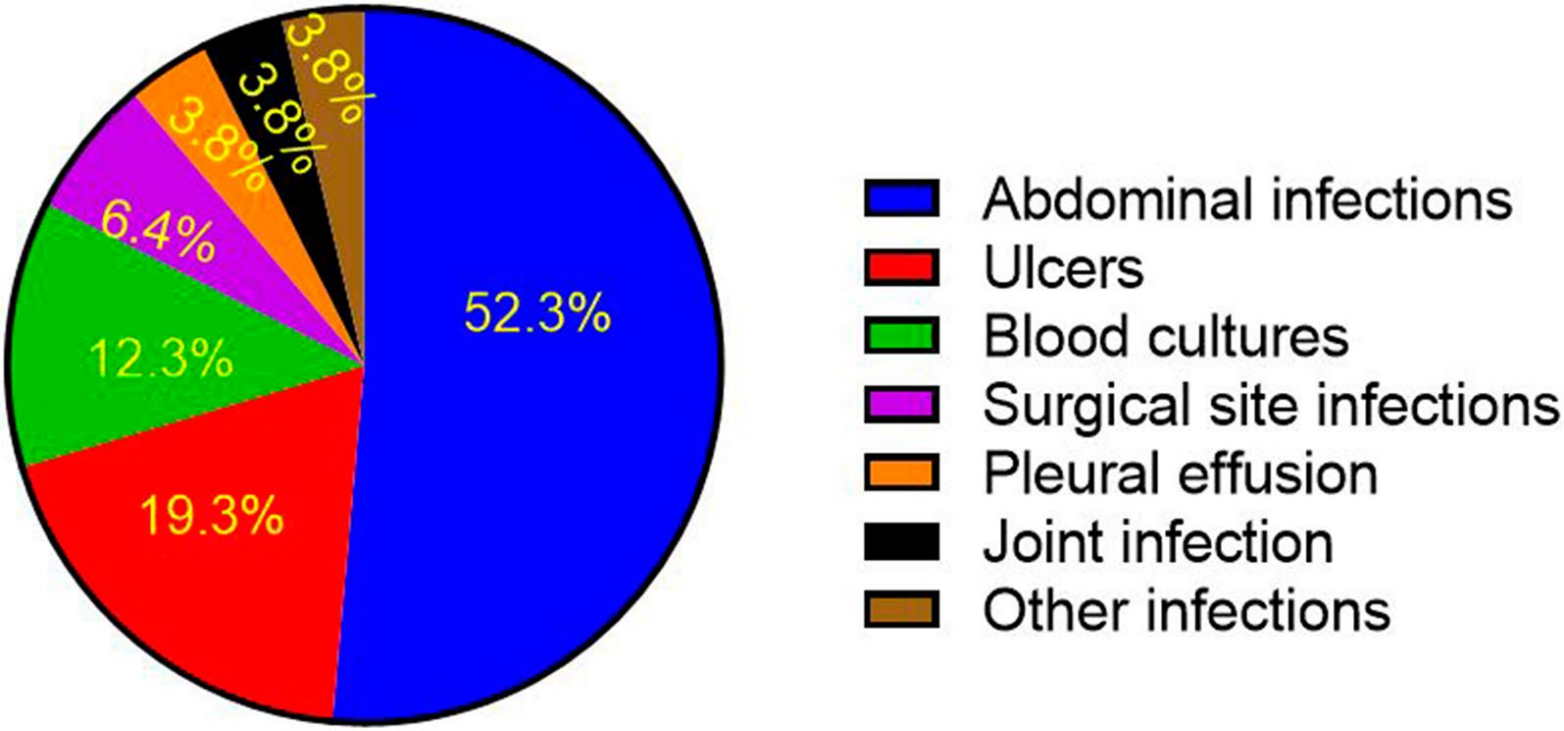
Clinical sample frequency according to clinical sample type

In this study, 130 clinical samples were collected in patient hospitalized in different part of the hospital. Frequency of samples according to clinical specimen type are shown in Fig. 1.

Cultivation results in 28 clinical samples (21.5%) were positive for anaerobic bacteria. The GenBank accession numbers of the 16 S rRNA gene for these bacteria were MN982885.1, MN955695.1, MN955694.1, MN955585.1, MN955548.1, MN955546.1, MN94720209.1, MN949555 M55.1, MN955544.1, MN954671.1, MN954561.1, MN954557.1, MN937266.1, MN937239.1, MN933933.1, and MN933926.1. *B. fragilis* (n = 22; 46.8%) was the most isolated species among 47 anaerobic bacteria. Table 1 shows the frequency of anaerobic bacteria isolated from clinical specimens.

**Table1:** Anaerobic bacteria isolated from clinical specimens

Anaerobic bacteria (Genus)	N (%)
*Bacteroides* spp.	
*Bacteroides fragilis*	22 (46.8)
*Bacteroides thetaiotaomicron*	3 (6.3)
*Bacteroides stercoris*	2 (4.3)
*Clostridium* spp.	
Clostridium *clostridioforme*	2 (4.3)
*Clostridium perfringens*	2 (4.3)
*Clostridium sporogenes*	1 (2.1)
*Paeniclostridium sordelli*	1 (2.1)
*Prevotella* spp.	
*Prevotella bivia*	2 (4.3)
*Prevotella oralis*	1 (2.1)
*Fusobacterium mortiferum*	1 (2.1)
*Veillonella* spp.	2 (4.3)
*Veillonella parvula*	2 (4.3)
Other *Veillonella* spp.	
Gram positive cocci	
* Anaerococcus prevotii*	1 (2.1)
*Finegoldia magna*	2 (4.3)
*Peptoniphilus asaccharolyticus*	1 (2.1)
*Peptostreptococcus* spp.	1 (2.1)
*Parvimonas micra*	1 (2.1)
Total	47 (100)

From 40 colorectal tissue biopsies in healthy individuals, 56 *B. fragilis* isolates were identified in 24 specimens (60%). Resistance patterns of the *B. fragilis* isolates, reporting MIC_50_ and MIC_90_, are shown in Table 2. The *B. fragilis* isolates had the highest resistance rate to penicillin (100%), tetracycline (74.4%), clindamycin (41%) and cefoxitin (38.5%).

**Table 2:** In vitro activities of nine antibiotics against isolated *B. fragilis*

Antibioticss	Patient Group (clinical samples)				All				Healthy Group (colorectal samples)			
	Range	MIC (µg/mL)		R%	Range	MIC (µg/mL)		R%	Range	MIC (µg/mL)		R%
		MIC_50_	MIC_90_			MIC_50_	MIC_90_			MIC_50_	MIC_90_	
Penicillin G	4->256	256	>256	100	4->256	128	> 256	100	4->256	128	> 256	100
Ampicillin-sulbactam	1–128	4	16	9.1	0.125–256	1	8	5.45.4	0.125–256	1	16	6.4
Piperacillin-tazobactam	0.06–256	1	64	9.1	0.06–128	0.5	4	1.8	0.06–256	0.4	4	2.6
Cefoxitin	2–256	16	256	45.5	2–256	8	256	35.7	2–256	16	256	38.5
Imipenem	0.06–4	0.5	4	0	0.064–16	0.5	4	1.8	0.06–16	0.5	4	1.3
Meropenem	0.064–4	0.25	2	0	0.064–16	0.25	2	1.8	0.06–16	0.1250.125	2	1.3
Tetracycline	1–128	32	64	81.8	0.125–128	32	64	71.4	0.125–128	32	64	74.4
Clindamycin	0.125->256	8	>256	54.5	0.125->256	2	256	42.9	0.125->256	16	256	41
Metronidazole	0.06–4	0.52	2	0	0.06–1	0.25	0.5	0	0.06–4	0.25	2	0

R %, Resistance percentages; MIC_50_ and MIC_90_, the minimum inhibitory concentration required to inhibit 50% and 90% of the bacteria population.

*tetQ*, *ermF*, *ermB*, *cfiA*, *cepA*, *cfxA*, *mefA*, *nim* genes and the insertion sequence IS1186 were further searched to evaluate antibiotic resistance by the PCR. Absolute and relative frequencies of resistance and insertion sequences genes are presented in Table 3.

*tetQ*, *ermF*, *ermB*, *cfiA*, *cepA*, *cfxA*, *mefA*, *nim* genes and the insertion sequence IS1186 were further searched to evaluate antibiotic resistance by the PCR. Absolute and relative frequencies of resistance and insertion sequences genes are presented in Table 3.

**Table 3:** Resistance genes and *bft* gene in *B. fragilis* isolates

Genes	Patient Group (clinical samples) N (%)	Healthy Group (colorectal samples) N (%)	All N (%)
*cfiA*	4 (18.1)	7 (12.5)	11 (14.1)
*cepA*	15 (68.2)	42 (75)	57 (73.1)
*cfxA*	5 (22.7)	15 (26.8)	20 (25.6)
*ermB*	0 (0)	0 (0)	0 (0)
*ermF*	16 (72.7)	34 (60.7)	50 (64.1)
*mefA*	2 (9.1)	3 (5.4)	5 (6.4)
*tetQ*	20 (90.9)	48 (85.7)	68 (87.2)
*nim*	0 (0)	0 (0)	0 (0)
*IS1186*	0 (0)	1 (1.8)	1 (1.3)
*bft*	5 (22.7)	10 (17.8)	15 (19.2)

In this study, *tetQ* (87.2%), *cepA* (73.1%) and *ermF* (64.1%) were the most abundant antibiotic-resistant genes. The *nim* and *ermB* genes were not detected in any of the isolates. The IS1186 sequence in the upstream region of the *cfiA* gene was detected in one isolate (1.3%); this isolate was also resistant to imipenem.

The presence of the *cfxA* and *ermF* genes were significantly higher in cefoxitin and clindamycin resistant isolates in compare with cefoxitin and clindamycin susceptible isolates (p = 0.001, 0.000).

**Fig. 2:**
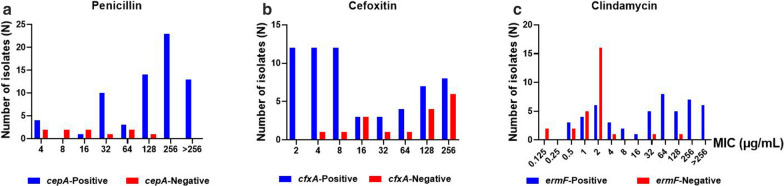
MIC values of (**a**) Penicillin, **b** Cefoxitin and **c** Clindamycin with the presence of the *cepA* gene, *cfxA* gene and *ermF* genes in *B. fragilis*

In addition, MIC values of penicillin, cefoxitin and clindamycin were significantly different among isolate with the *cepA*, *cfxA* and *ermF* genes in compare with isolates lacking these genes (p = 0.002, 0.000, 0.001) (Fig. 2).

In this study, the *bft* gene was observed in 22.7 and 17.8% of the clinical and colorectal isolates, respectively (Table 3).

## Discussion

*Bacteroidetes* as a large community of gut microbiota can be isolated from human clinical specimens and lead to mixed anaerobic bacterial infections [3]. Antibiotic-resistant genes also play important roles in the antibiotic resistance of *B. fragilis* and cause unsuccessful antibacterial therapy. In this study, we have evaluated the prevalence of resistance genes and antibiotic resistance profile of *B. fragilis* using phenotypic approaches and amplification of genes of interest.

In this study, *B. fragilis* accounted for 46.8% of anaerobic bacteria isolated from clinical samples.

The MIC_50_ and MIC_90_ values for ampicillin/sulbactam, piperacillin/tazobactam, metronidazole and clindamycin in clinical isolates were at least twice higher than colorectal isolates. One possible reason for this might be the use of antibiotics in these patients.

Although carbapenems have been considered as highly effective antibiotics in the prevention of anaerobic infections, bacterial resistance to these antibiotics has increased [8, 12, 16, 28]. In this study, 1.3% of isolates (n = 1) were resistant to imipenem and 1.3% of isolates (n = 1) were resistant to meropenem. These isolates were collected from the GIT of healthy individuals which could be considered as a serious risk for public health. The emergence of carbapenem resistance has also been reported in different studies. For instance, meropenem resistance was found to be 0.5% in the United States and 2% in Europe [8, 13, 29]. In a study conducted by Kohsari et al. in Iran, the resistance of *B. fragilis* to meropenem was 13.9% [30]. Discrepancies observed in different studies regarding antibiotic resistance profile of *B. fragilis* may be due to different reasons including geographical features, population study, and differences in laboratory techniques.

Resistance to carbapenems in *B. fragilis* is usually caused by the expression of the class B metallo-beta-lactamase encoded by the *cfiA* gene, located on the chromosome. Accordingly, if an insertion sequence is located in its upstream region, the gene will be expressed and will cause carbapenem resistance [4, 31]. In a study conducted by Sóki et al. 11 out of 15 *cfiA* positive *B. fragilis* isolates were resistant to imipenem [32]. In the present study, 18.1 and 12.5% of the clinical and GIT samples had the *cfiA* gene, respectively. Moreover, the imipenem-resistant isolates had the *cfiA* gene and the IS1186 insertion sequence in the upstream region of the gene whereas the meropenem-resistant strain had this gene but lacked the IS1186 insertion sequence. The resistance was possibly due to expression of the silent carbapenemase gene [33], the presence of other insertion sequences in the upstream region of this gene (IS1187, IS1188, IS942) [29], or other resistance mechanisms such as membrane permeability or penicillin-binding protein (PBP) affinity [34]. In addition, some isolates had the *cfiA* gene but were phenotypically sensitive to carbapenem which demonstrate the antibiotic resistance gene may not be expressed. In a study performed by Rashidian et al. in Iran, 31.5 and 20% in *B. fragilis* group isolate from the patients and control groups harbored *cfiA* gene, respectively [35].

Penicillins and second-generation cephalosporin resistance have also been observed in *B. fragilis*.

The most important mechanisms contributing to this resistance is the expression of beta-lactamases which are encoded by the *cepA* gene (resistance to penicillin and cephalosporins other than cefoxitin) and *cfxA* gene (resistance to cefoxitin) [36, 37]. In this study, all the isolates (100%) were resistant to penicillin, of which 73.1% had the *cepA* gene. There was also meaningful difference in penicillin MIC value of isolates with *cepA* gene compared to isolates without *cepA* gene indicating the importance of this gene in resistance to penicillin. In addition, 45.5 and 35.7% of the clinical and colorectal isolates were respectively resistant to cefoxitin, and 22.7 and 26.8 % of these isolates had the *cfxA* gene, respectively. The presence of the *cfxA* gene was significantly higher in cefoxitin-resistant isolates compare to cefoxitin- susceptible isolates, which was also statistically significant.

The rate of *B. fragilis* resistance to cefoxitin in recent years has been 6.8–33.3% in Europe, 12.6% in Canada, and 23% in Brazil [8, 38, 39]. In a study conducted by Kangaba et al. in Turkey, 28% of *B. fragilis* isolates and 32% of isolates from the GIT had been found to be resistant to cefoxitin. In this study, resistance to ampicillin/sulbactam and piperacillin/tazobactam were 6.4 and 2.6%, respectively [6]. In another investigation, 5.4% of *B. fragilis* isolates were resistant to piperacillin/tazobactam which was relatively consistent with the findings reported by Maraki et al. (5.%) and Yunoki et al. studies (2.8%) [16, 40].

The *ermB* and *mefA* genes were also involved in the development of macrolide resistance in *B. fragilis* [41]. The prevalence of clindamycin resistance had been further reported by 54.5% in clinical isolates and 42.9% in the colorectal isolates which were mainly associated with the presence of the *ermF* gene [37]. Clindamycin resistance among *B. fragilis* have been reported in several countries [10, 42–44].

In the present study, all clindamycin-resistant isolates had the *ermF* genes. In addition, five isolates had the *mefA* gene and three of which were clindamycin-resistant strains. The presence of the *ermF* gene also was higher in clindamycin-resistant isolates than clindamycin susceptible-isolates respectively, which was statistically significant. None of the isolates in this study had *ermB* gene.

The presence of *tetQ* gene associated with tetracycline resistance has been further reported in clinical isolates [40, 45]. In the present study, 81.8 and 71.4 % of the clinical and colorectal isolates had tetracycline resistance, and 90.9 and 85.7% of these isolates had the *tetQ* genes, respectively.

In a study conducted by Narimani et al. 86% of the colorectal isolates were resistant to tetracycline, and the *tetQ* gene was found in 85% of the isolates [45]. In the investigation by Kangaba et al. study, 72% of clinical isolates and 92% of colorectal isolates were resistant to tetracycline, 64 and 92% of them had the *tetQ* gene, respectively [6].

The metronidazole resistance rate was found to be 0–3% in different parts of the world [6, 8, 35, 46]. In addition, Different rates of resistance to metronidazole were reported in different part of Iran. Both in our study and in studies by Rashidan et al., all evaluated strains were susceptible to metronidazole and none of the strains contained *nim* genes [35]. In a study conducted by Akhi et al. in west of Iran, 8 (32%) out of 26 *B.fragilis* group isolated from Surgical site infection were resistant to metronidazole [47]. In another study performed by Kouhsari et al. in center of Iran, 6 (1.2%) out of 475 *B.fragilis* group isolated from Surgical site infection were resistant to metronidazole [30]. This difference in results, may cause from variation in antibiotic usage history of patients, geographical region, and sample size. However, future studies are needed to confirm these results in a higher sample collection from different provinces of Iran.

Based on previous studies, the prevalence of the *bft* gene was reported to be 6.2–20% in the colorectal isolates [34, 48–51] and 18.5–38.2% in clinical isolates [51–53] which was consistent with the findings in the present study.

Although phenotypic findings indicated resistance to some antibiotics in this study, the PCR findings did not confirm the presence of corresponding resistance genes in the isolates. This fact may suggest the role of other resistance mechanisms such as efflux pumps, changes in the cell wall structure, and catalytic enzymes in *B. fragilis* isolates [37, 54] that need further investigation.

## Conclusions

In our study, metronidazole was only the most *in vitro* active agent against all of *B. fragilis* isolates and should be considered as a first-line antibiotic for the empirical treatment of *B. fragilis* infection. It was concluded that continuous monitoring of antibiotic resistance patterns of *B. fragilis* in different geographical areas was crucial to provide a suitable treatment profile and to prevent infection more accurately. In addition, with regard to the presence of antibiotic-resistant genes and the high risk of antibiotic-resistant strains in the GIT of healthy people, proper prescription of antibiotics and avoidance of its arbitrary use can help prevent infection and transmission of resistant isolates.

## Data Availability

All data relevant to the study are included in the article.

## Abbreviations

*B. fragilis*: *Bacteroides fragilis*
GIT: Gastrointestinal tract
ETBF: Enterotoxigenic *B. fragilis*
BFT: *B. fragilis* toxin
IBS: Irritable bowel syndrome
CRC: Colorectal cancer
CLSI: Clinical and Laboratory Standards Institute
MIC: Minimum Inhibitory Concentration
CFU: Colony-forming unit
BBE: Bacteroides Bile Esculin Agar
BBA: Brucella Blood Agar
PCR: Polymerase Chain Reaction
